# Deep learning-based quantification of eosinophils and lymphocytes shows complementary prognostic effects in colorectal cancer patients

**DOI:** 10.1038/s41698-025-00955-0

**Published:** 2025-06-13

**Authors:** Elias Baumann, Sophie Lechner, Philippe Krebs, Richard Kirsch, Martin D. Berger, Alessandro Lugli, Iris D. Nagtegaal, Aurel Perren, Inti Zlobec

**Affiliations:** 1 Institute of Tissue Medicine and Pathology, University of Bern, Bern, Switzerland; 2 Graduate School for Cellular and Biomedical Sciences, University of Bern, Bern, Switzerland; 3 Pathology and Laboratory Medicine, Mount Sinai Hospital, University of Toronto, Toronto, Ontario Canada; 4 Department of Medical Oncology, Inselspital, Bern University Hospital, University of Bern, Bern, Switzerland; 5 Department of Pathology, Radboud University Medical Center, Nijmegen, The Netherlands

**Keywords:** Cancer microenvironment, Colorectal cancer, Prognostic markers

## Abstract

The immune microenvironment of colorectal cancer is a major component of the disease and influences not only tumor progression and patient outcome but also therapy response. Expanding on existing studies which have explored the prognostic value of the adaptive immune response with lymphocytes, our study integrates innate immune cells, specifically eosinophils, in a combined analysis. To evaluate the prognostic significance of eosinophils within the context of lymphocyte infiltration, we analyzed a large collective of 1625 colorectal cancer cases from four different centers. For this purpose, we develop an automatic deep learning pipeline for quantification of these immune cells directly from hematoxylin and eosin-stained whole slide images. Our analysis shows eosinophils in the tumor front (EosF) as independent prognostic factor (HR = 0.70, 95%CI = 0.55 − 0.90, *p* = 0.005), particularly also in microsatellite instability (MSI) cases (HR = 0.32, 95%CI = 0.14 − 0.74, *p* = 0.008). Moreover, EosF and intraepithelial lymphocytes (IELs) counts are statistically independent and provide additive prognostic information (EosF: HR = 0.71, 95%CI = 0.55 − 0.90, *p* = 0.005, IELs HR = 0.59, 95%CI = 0.35 − 0.99, *p* = 0.047). Our study demonstrates that eosinophils are an independent prognostic factor, which can be automatically quantified, underscoring its high potential for translation to a diagnostic biomarker. Moreover, our work could pave the way towards an integrated immune score directly from hematoxylin and eosin-stained sections.

## Introduction

Cancer is a complex disease defined not just by malignant cells themselves but also the surrounding cells, molecular signals and microbiome. The tumor microenvironment (TME) is a central component of disease progression and its constituents, including immune cells, define whether the TME has a pro- or anti-tumor function^1^. Lymphocytes have demonstrated prognostic value, both when assessed via hematoxylin & eosin (H&E)-stained tissue slides^2^, or via immunohistochemistry (IHC) for specific subsets such as CD3+, CD8+ or FOXP3+ lymphocytes^3^. Galon et al.^4^ then integrated multiple T-cell subsets into an Immunoscore®, which has applications in colorectal cancer (CRC). As counting cells manually is time consuming, global descriptions of immune response such as a common 3-tiered description (“hot”,“cold”,“excluded”) have been defined. However, these descriptions focus on the most abundant cell populations present within the tumor, namely lymphocytes, and ignore other immune cells^5^, leaving a gap in knowledge with regards to the clinical relevance of less-studied immune cell populations. Moreover, as shown in the Immunoscore®, and other studies investigating the TME, separately quantifying immune responses in the tumor front or within the tumor center, or separating the intratumoral stromal compartment from the intra-epithelial lymphocytes impact prognosis differently^3,4^. These results suggest that the location of these cells should be taken into account in the assessment of immune components.

In clinical practice, treatment decisions for stage II colon cancer patients regarding adjuvant chemotherapy depend on disease aggressiveness and microsatellite instability (MSI) status and require biomarkers for improved decision-making^6^. MSI is important to help select patients with metastatic CRC for possible immunotherapy regimens^7^ and MSI cases are generally more inflamed than microsatellite stable (MSS), indicating that they should therefore be investigated separately as well. At the molecular level, classification systems such as the consensus molecular subtypes (CMS)^8^ and pathway-derived subtypes (PDS)^9^ categorize CRCs based on bulk RNA-seq profiles. Strong correlation with these subtypes could indicate a link between molecular profiles and specific immune cells. Eosinophils, although frequently co-occurring with lymphocytes^10,11^, remain understudied in CRC. While primarily researched in allergy and asthma, eosinophils also play a role in wound healing and tissue repair^12^, a physiological process likened to the earliest stage of cancer^13^.

In the healthy colon, recent research has identified two distinct eosinophil types (basal, active) with different functional states^14^. In CRC, experimental studies suggest different roles for eosinophils in the anti tumor response in CRC, either having a direct cytotoxic role stimulated by IFNG^15,16^ or an indirect role via the recruitment of cytotoxic T-cells^17^. Studies investigating the abundance of eosinophils in CRC as a prognostic factor show mixed results in particular when adjusting for disease stage^18^. Questions regarding the prognostic relevance of eosinophils, their frequency, location and correlation with other tumor parameters remain open. In fact, still missing is an in-depth characterization of CRCs with eosinophils, and the potential clinical and prognostic value of eosinophils for patients, in an appropriately powered cohort.

One reason for this may be the laborious and time-consuming quantification of cell types from H&E slides based on morphology, which can now be overcome using deep learning methods. Deep learning models are trained and validated on large cohorts of patient data to then automatically identify, classify and quantify cells^19^ and tissue types^20^. The aim of this study is to perform an in-depth investigation of the frequency and distribution of eosinophils in CRC, their correlation with tumor features and patient outcomes, as well as their potential interaction with lymphocytes. To do so, we establish deep learning algorithms for the detection and quantification of immune cell populations from routine H&E-stained whole slide images, from 1625 patients with 2529 slides across four independent retrospectively collected cohorts. Our study demonstrates that eosinophil counts in the tumor front are an important prognostic marker in CRC across all disease stages, and in MSI CRCs with higher levels indicating a more favorable prognosis. Next, we found that IELs and eosinophils have no statistical relationship and provide additive prognostic value indicating that eosinophil levels may be indicators of a different and unrelated molecular process of anti-tumor activity.

## Results

### HoVer-NeXt can accurately identify lymphocytes, eosinophils and IELs in H&E WSI

The nuclei segmentation and classification model HoVer-NeXt has previously been extensively validated and reached an F1 score of 0.77 for lymphocyte detection on the test-set, and 0.83 for epithelial cells^19^. Compared to epithelial cells and lymphocytes, eosinophils are rare, and to ensure that eosinophil detections using HoVer-NeXt are accurate, a manually exhaustively annotated test-set was created. Consisting of eleven regions of interest (ROIs) from seven whole slide images (WSI) with a total of 4952 eosinophils, eosinophils with a visible nucleus were exhaustively annotated within each ROI. Eosinophilic granules without a visible nucleus were not considered, as the model can not detected cells without a visible nucleus. HoVer-NeXt reaches a F1 score of 0.73 ± 0.07 with a precision of 0.71 ± 0.12 and a recall of 0.78 ± 0.07. Additionally, IELs were validated on the same set of ROIs reaching an F1 score of 0.407 ± 0.061 and a ROI level correlation of *ρ* = 0.87. The dataset is available at https://zenodo.org/records/11657620.

### Eosinophils and IELs are Independent Aspects of the Tumor Immune Microenvironment

**Fig. 1:**
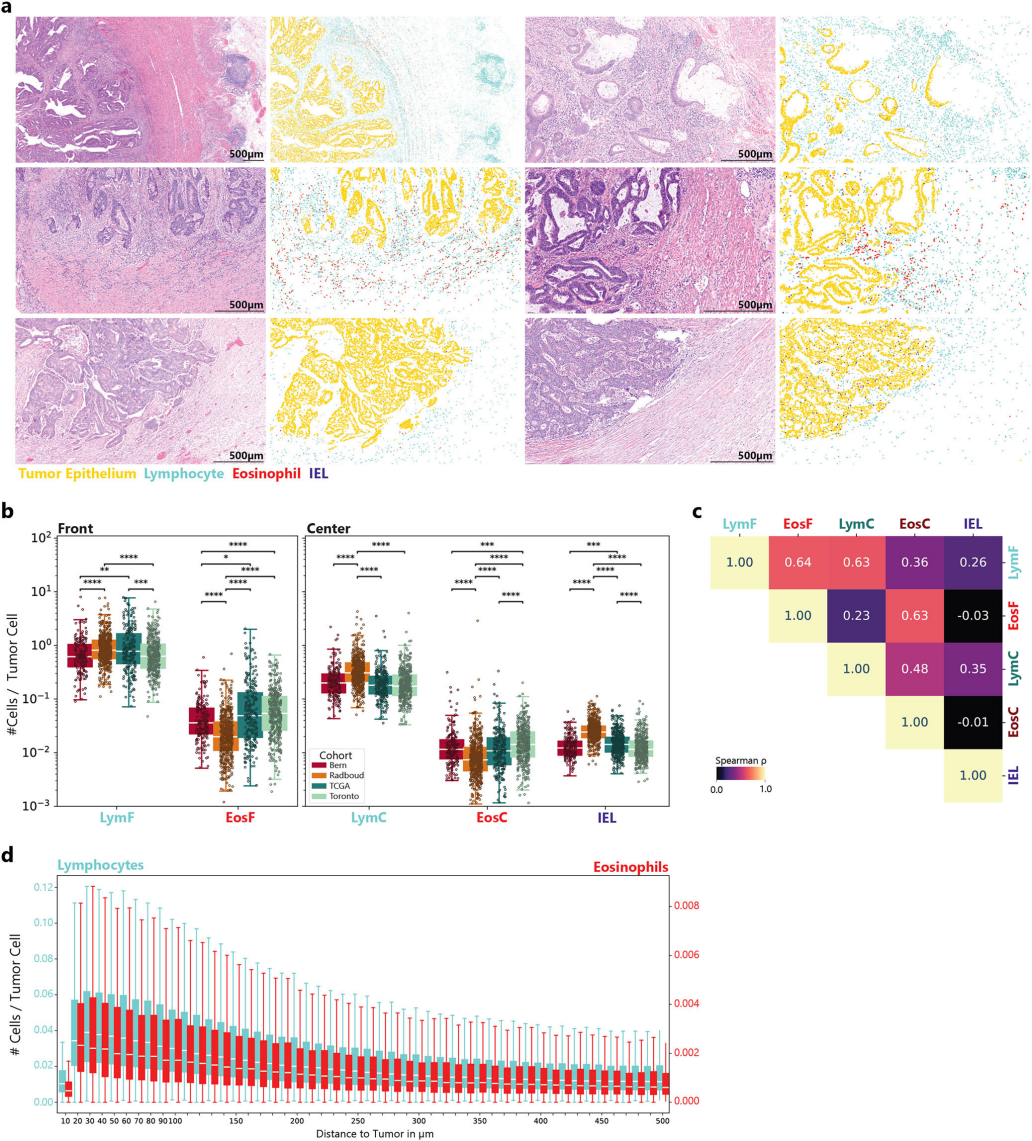
Qualitative and descriptive data analysis. **a** Visual examples of selected representative different immune compositions with predicted locations of tumor epithelium, lymphocytes, eosinophils and IELs. The top row shows two cases with strong lymphocytic immune response, even with IELs. The second row shows two cases with high eosinophil to lymphocyte ratio and the third row two cases with low immune response, but one without many IELs and one with many IELs (right). **b** Abundance of immune cells in different cohorts normalized by number of tumor epithelial cells. **c** Rank correlation between different immune subsets. **d** Number of cells per tumor cell within distance to tumor binned into 10*μm* bins. *P*-Values below 0.05 are considered significant (* < 0.05, ** < 0.01, *** < 0.001, **** < 0.0001), box plots show whiskers spanning from min to max for values within 1.5 × *IQR*.

In a first step, we examine the frequency, distribution and location of eosinophils, lymphocytes and IELs to investigate their correlations and potential interactions. Figure 1a shows exemplary regions of interest with different immune infiltration. We analyze the abundance of the selected immune cell groups in the respective regions, normalized by the number of tumor epithelial cells. On average, we find more eosinophils and lymphocytes in the tumor front (EosF, LymF) than center (EosC, LymC) though with similar relative abundances (EosF: 6.02% ± 4.49%, EosC: 5.72% ± 3.93%). Cohort specific differences across all quantified cell types can be observed. Therefore, in all following experiments, scores are percentile normalized per cohort. We use the fifth and 95th percentile of stage II cases as “minimum” and “maximum” for min-max scaling to have the same range for all datasets but stay robust to outliers. These outliers often arise in cases with low tumor cell count, and subsequently receive a value above 1.0 after normalization, but are still included in the analysis without heavily impacting regression results. Lymphocytes and eosinophils are significantly correlated, particularly in the tumor front (*ρ* = 0.64). LymF and LymC (*ρ* = 0.63) as well as EosF and EosC (*ρ* = 0.63) are positively correlated. In contrast, only a weak correlation was observed between IELs and LymC (*ρ* = 0.35), and no correlation between IELs and eosinophils (EosF *ρ* = 0.03, EosC *ρ* = − 0.01).

Using the Bern cohort with multiple slides for each patient, we analyze intra-patient heterogeneity and find substantial differences between slides for all three cell types (Supp. Fig. S3). For LymF, approximately 20% of cases have a single slide with more than three times the amount of cells than the median of that same case. These inter-slide variations emphasize the importance of investigating the spatial distribution of immune cells within the tumor microenvironment which could have further functional implications. Figure 1d shows the tumor epithelial cell normalized lymphocyte and eosinophil counts binned into distances to the next tumor epithelial cell (bin size = 10*μm*). We can observe that for both lymphocytes and eosinophils, the highest abundance is within close proximity to tumor cells (Lymphocytes: *r* = 20 − 30*μm*, Eosinophils: *r* = 10 − 20*μm*). Both show a declining median and shrinking inter quartile range towards 500*μm*. Most of the per case abundance, but also between case variance is therefore in close proximity of tumor cells. One potential confounder for increased eosinophilic inflammation in the tumor could be a higher level of inflammation in the corresponding normal tissue. In a subset of patients with available adjacent normal tissue slides (*N* = 52) we therefore compare immune cell quantities. In all three immune populations (eosinophils, lymphocytes and IELs), no significant correlation between the numbers of these cell types in normal mucosa and their respective colon cancer was observed (Supp. Fig. S2).

Summarizing, per patient differences in immune response are primarily driven by eosinophils and lymphocytes close to the tumor. Within a limited subset, our results could not show a correlation between eosinophils in the normal adjacent mucosa and the prevalence of eosinophils in close proximity of the tumor. Finally, while EosF and LymF show some correlation, EosF and IELs are statistically independent aspects of the immune response.

### EosF are Correlated with Favorable Prognostic Factors

**Fig. 2:**
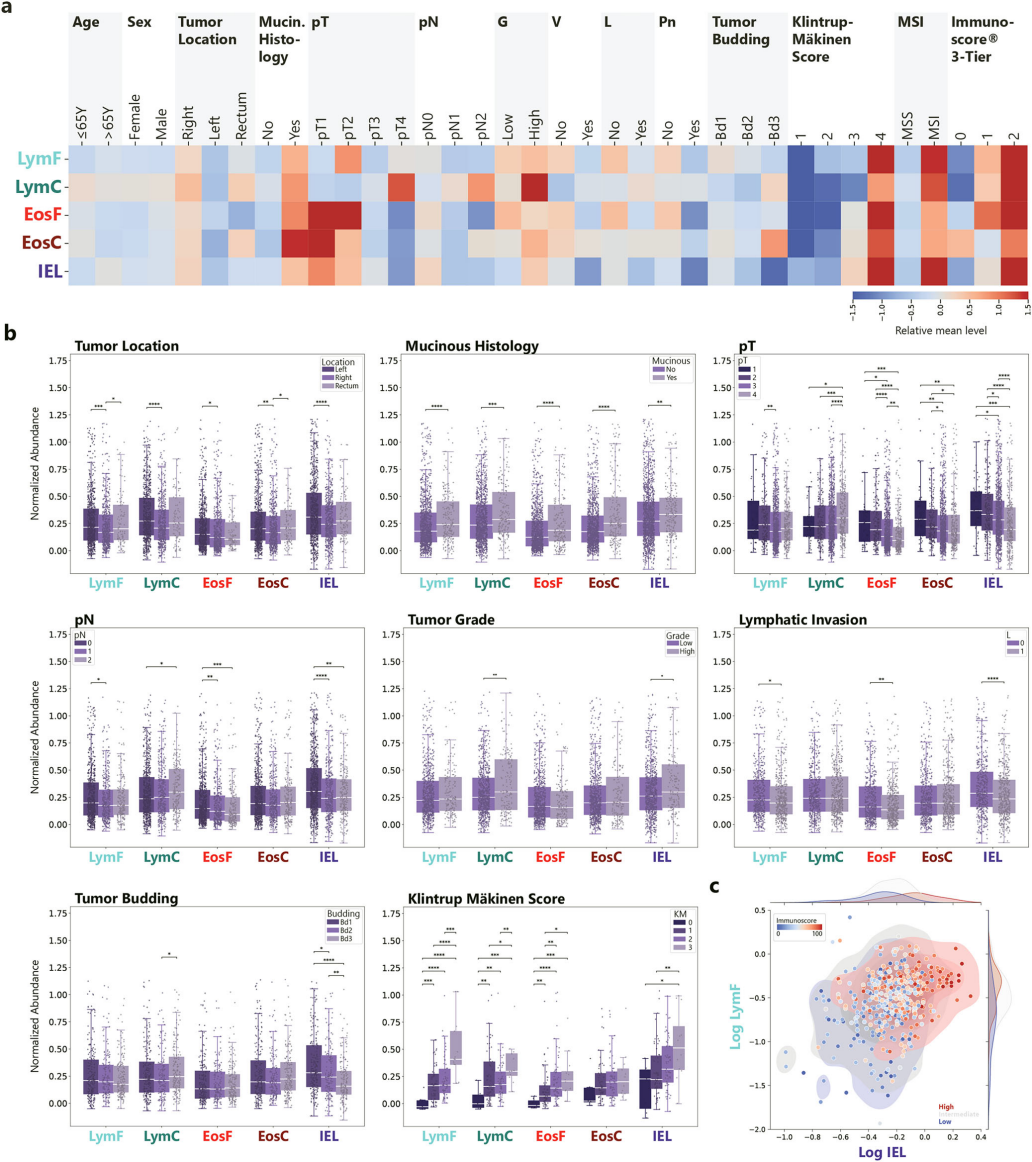
Association with clinicopathological characteristics. **a** Heatmap overview with column normalized score levels. **b** Selected characteristics with comparison of abundance levels of different cell groups. **c** Association with Immunoscore®. *P*-Values below 0.05 are considered significant (* < 0.05, ** < 0.01, *** < 0.001, **** < 0.0001), box plots show whiskers spanning from min to max for values within 1.5 × *IQR*.

To investigate the prognostic significance of these immune sub-populations, we first evaluate the correlation and association with clinicopathological characteristics. An initial look at all correlations on the heatmap shows some similarities between all immune groups across the different variables (Fig. 2a). Regarding clinical characteristics, all groups are significantly enriched in right sided tumors (*p* < 0.05), but there is no significant associations with age or sex. For tumor stage, EosF and IELs levels decrease with increasing pT and pN (*τ* < − 0.09, *p* < 0.001).

In contrast, LymC show higher abundance in higher pT (*τ* = 0.09, *p* < 0.001) and also in higher tumor grade (*p* < 0.01) where detected IELs are also slightly more abundant (*p* < 0.05). For markers of invasion, such as lymphatic invasion, venous invasion or perineural invasion, EosF and IELs are higher in cases without invasion (*p* < 0.01). Only IELs are higher in cases with lower tumor budding (*p* < 0.001). All investigated immune cell groups except for eosinophils in the tumor center correlate with increasing Klintrup-Mäkinen score, (LymF *τ* = 0.33, *p* < 0.001, EosF *τ* = 0.31, *p* < 0.001, IEL *τ* = 0.24, *p* = 0.001). The Immunoscore® is highly correlated with IELs (*ρ* = 0.48, *p* < 0.001), moderately with LymF (0.34, *p* < 0.001) and only slightly with EosF (0.14, *p* = 0.002).

While all immune cells are enriched in right-sided tumors, EosF and IELs specifically associate with features of less aggressive disease, including lower stage and absence of invasion. EosF, LymF and IELs show varying degrees of correlation with established inflammation markers like Klintrup-Mäkinen score. This leads to the question whether eosinophil presence could be driven by the same molecular program as MSI.

### CMS, PDS and MSI do not Explain the Presence of Eosinophils

**Fig. 3:**
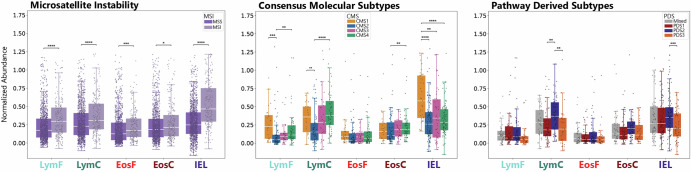
Correlation with MSI and additional molecular subtypes. Comparison of abundance levels of different cell groups in the respective subtypes. *P*-Values below 0.05 are considered significant (* < 0.05, ** < 0.01, *** < 0.001, **** < 0.0001), box plots show whiskers spanning from min to max for values within 1.5 × *IQR*.

MSI CRCs exhibit higher neoantigen loads^21^ and higher infiltration with lymphocytes and cytotoxic lymphocytes specifically^22^. In line with these reports, in MSI cases, we find significantly higher amounts of lymphocytes and eosinophils in front and center, as well as IELs (EosC *p* = 0.049, others *p* < 0.001). CMS and PDS represent different molecular configurations based on clustering gene expression or pathway enrichment levels. A correlation with one of the subtypes could indicate a relationship between the immune cell prevalence and the molecular properties of the tumor. Immune-enriched CMS1 and stromal CMS4 show significant enrichment of LymF and LymC compared to CMS2 (*p* < 0.01). IELs are enriched in CMS1 compared to all other groups (*p* < 0.01). EosF are not significantly different in any of the categories, EosC are lower in CMS2 compared to CMS4 (*p* < 0.01). For PDS, PDS2 is enriched for LymC (*p* < 0.01), and PDS3 has lower IELs compared to PDS2 (*p* < 0.001) (Fig. 3). In summary, in line with previous work, lymphocytes and IELs are predominantly associated with CMS1, CMS4, PDS2 (Fig. 3), and MSI CRCs (Fig. 2). Eosinophils are also enriched in MSI tumors, although not overrepresented in any of the CMS or PDS subtypes.

### EosF, LymF and IELs are Prognostic in Univariate Survival Analysis

**Fig. 4:**
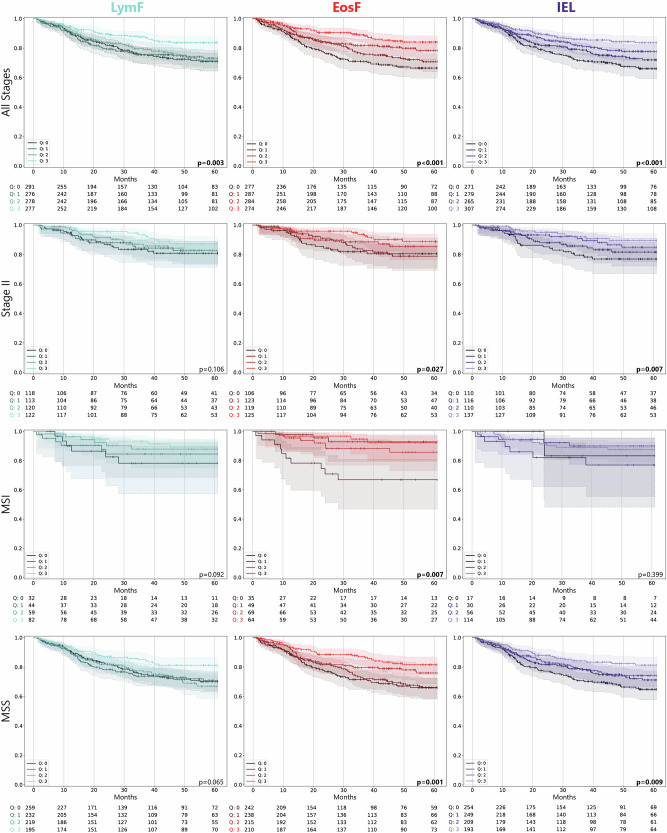
KM plots for univariate survival analysis. KM plots for three selected immune groups: LymF, EosF and IEL with TTR as endpoint. For visualization purposes, continuous scores are split into quartiles based on the full set of samples. The first row shows the KM plots for all cases, the second row only for stage II, the third row only for MSI cases, and the fourth row for MSS Cases. *P*-values are Wald-tests against the lowest quartile.

**Fig. 5:**
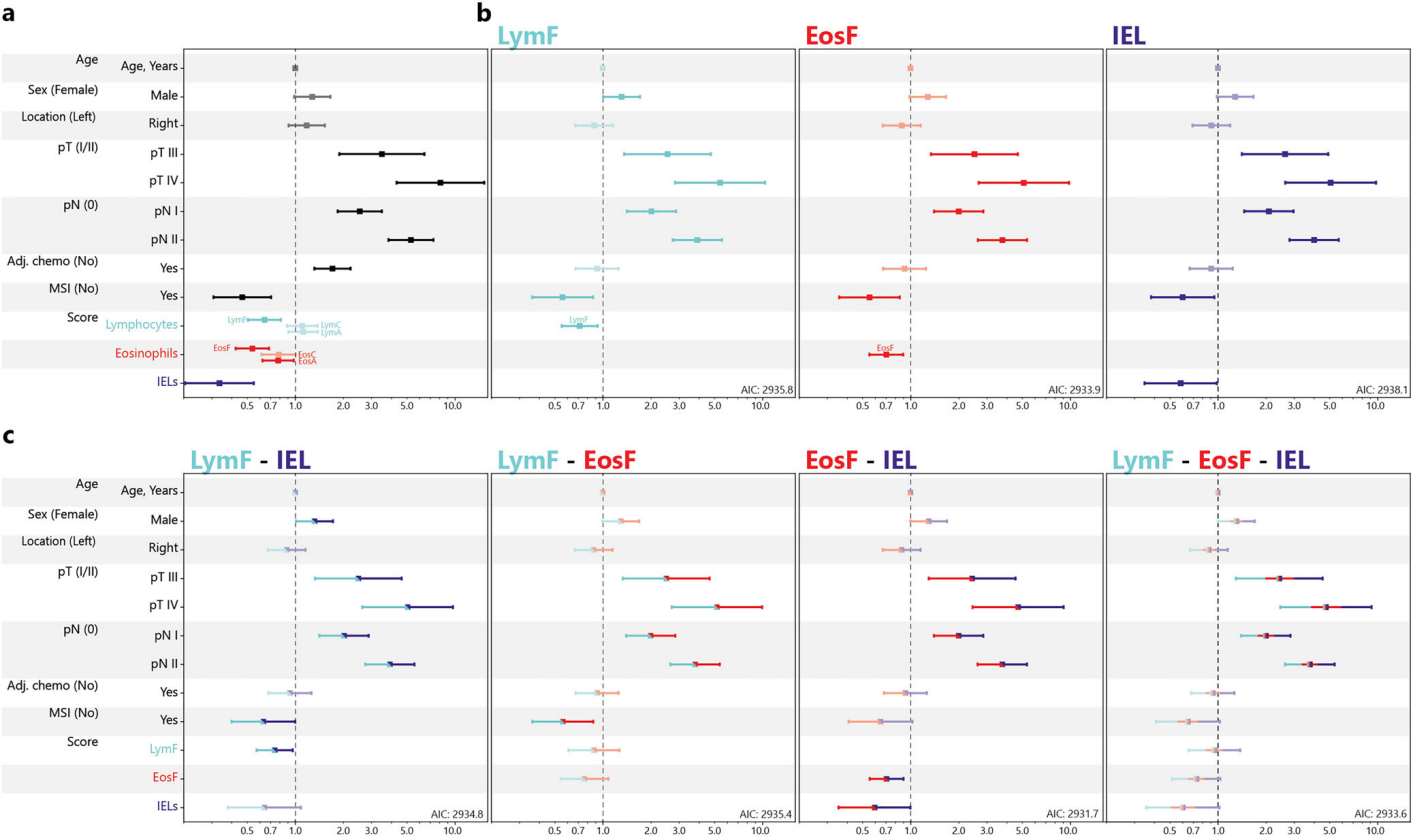
Survival analysis (TTR) with Cox proportional hazard models. **a** Univariate analysis with each variable later to be used in the multivariate models. Confidence intervals crossing the 1.0 hazard ratio line indicate a non-significant variable. **b** Multivariate analysis using a single immune groups (LymF, EosF, and IEL). **c** Combined analysis with multiple immune groups in the same model: LymF + IEL, LymF + EosF, EosF + IEL, and LymF + EosF + IEL.

Using KM estimators and quartile splits, we find significant differences and more favorable survival with higher values of LymF, EosF and IELs across all stages, whereas in Stage II, only EosF and IELs are associated with improved outcome. In MSI CRCs, low eosinophil counts are associated with unfavorable survival, whereas in MSS CRCs, again IELs and EosF show significant differences in survival with higher values again indicating more favorable survival (Fig. 4). Using Cox regression analysis, we investigate the impact of immune cell type as a continuous score on TTR. Considering CRCs of all Stages (*N* = 1122, 229 events), LymF (HR = 0.64, 95%CI = 0.51 − 0.81, *p* < 0.001), EosF (HR = 0.54, 95%CI = 0.42 − 0.68, *p* < 0.001), IELs (HR = 0.34, 95%CI = 0.20 − 0.55, *p* < 0.001) and EosA (HR = 0.78, 95%CI = 0.62 − 0.98, *p* = 0.03) are significantly associated with TTR, all with HR<1 indicating a positive prognostic effect. After assessing the goodness-of-fit of all variables using the Akaike information criterion (AIC), and to avoid statistical issues of multi-co-linearity, we settle on EosF, LymF and IELs for further analyses. In Stage II (*N* = 474, 64 events), again in univariate analysis, the LymF (HR = 0.62, 95%CI = 0.40 − 0.98, *p* = 0.039), EosF (HR = 0.61, 95%CI = 0.39 − 0.95, *p* = 0.028), and IELs (HR = 0.25, 95%CI = 0.09 − 0.67, *p* = 0.006) indicate a better prognosis with increased prevalence (Supp. Tab. S4). For MSI CRCs (*N* = 217, 25 events), EosF are significant (HR = 0.34, 95%CI = 0.16 − 0.76, *p* = 0.008), whereas LymF (HR = 0.50, 95%CI = 0.24 − 1.05, *p* = 0.065) and IELs (HR = 0.72, 95%CI = 0.26 − 1.98, *p* = 0.524) are not (Supp. Tab. S6). Finally, for MSS CRCs (*N* = 905, 204 events), in univariate analysis, LymF (HR = 0.72, 95%CI = 0.56 − 0.93, *p* = 0.010), EosF (HR = 0.6, 95%CI = 0.47 − 0.77, *p* < 0.001), and IELs (HR = 0.35, 95%CI = 0.19 − 0.63, *p* < 0.001) all have a positive prognostic effect in this subset. An overview of the results as a forest plot can be found in Fig. 5.

Summarizing, higher levels of LymF, EosF, and IELs are associated with a favorable prognosis across CRC stages and in stage II specifically. In MSI CRCs, only EosF is significant, yet all immune groups need further assessment in multivariate models including other clinicopathological characteristics.

### EosF and IELs are Independent Prognostic Factors with Additive Prognostic Value

LymF, EosF, and IEL, all correlate with established prognostic markers, and their independent contribution to outcome should be addressed. To do so, multivariate models including age, sex, tumor location, pT, pN, adjuvant chemotherapy status, and MSI status as covariates are examined, along with these three immune groups, alone and together. All covariates are included regardless of their univariate significance, as their impact may emerge in specific subgroups during multivariate modeling. When analysed individually, each of the three immune groups LymF (HR = 0.71, 95%CI = 0.55 − 0.92, *p* = 0.010), EosF (HR = 0.70, 95%CI = 0.55 − 0.90, *p* = 0.005) and IELs (HR = 0.58, 95%CI = 0.35 − 0.98, *p* = 0.043) are independent prognostic factors, with higher amounts of each cell type linked to favorable outcome. Including multiple cell types into combined multivariate analysis shows that IELs and EosF remain significant (EosF HR = 0.71, 95%CI = 0.55 − 0.90, *p* = 0.005, IELs HR = 0.59, 95%CI = 0.35 − 0.99, *p* = 0.047) in the same model. Moreover, analysing the goodness-of-fit of the various Cox regression models shows that the combined model of IELs and EosF has the lowest AIC value, indicating a better model fit (AIC values in Fig. 5). Subgroup analysis of stage II patients shows that none of the immune groups significantly contribute independent prognostic information after adjusting for all other covariates as possible confounders (LymF (HR = 0.64, 95%CI = 0.40 − 1.03, *p* = 0.068), EosF (HR = 0.67, 95%CI = 0.43 − 1.05, *p* = 0.084), IELs (HR = 0.40, 95%CI = 0.14 − 1.15, *p* = 0.090) (Supp. Tab. S5). While, EosF (HR = 0.32, 95%CI = 0.14 − 0.74, *p* = 0.008) maintain their prognostic information in MSI cases, all three scores are linked to prognosis in MSS cases (LymF HR = 0.74, 95%CI = 0.56 − 0.97, *p* = 0.030, EosF HR = 0.75, 95%CI = 0.58 − 0.97, *p* = 0.027, IEL HR = 0.51, 95%CI = 0.28 − 0.94, *p* = 0.030). Evaluating combinations of multiple scores in one model, however, leaves only EosF and IELs as combined independent prognostic factors (EosF HR = 0.76, 95%CI = 0.59 − 0.98, *p* = 0.033, IEL HR = 0.53, 95%CI = 0.29 − 0.96, *p* = 0.037). Full results can be found in supplementary Tables S3 (all stages), S5 (stage II), S7 (MSI), and S9 (MSS).

## Discussion

In this large scale, fully automated deep learning-based analysis we show that eosinophils in the tumor front are an independent prognostic factor, even providing further prognostic information for TTR when including IELs.

Previous studies have investigated the prognostic relevance of eosinophils in CRC^10,11,23–28^, but most of these studies rely on manual counting, in specific fields of view. In our study, we automatically count all eosinophils, and separate tumor front and tumor center, showing that eosinophils in the tumor front, while correlated with those in the tumor center, have more prognostic relevance. Väyrynen et al.^11^ automatically quantify eosinophils but perform their primary investigation on tissue microarray cores, which depend on preselecting region of interest as well as on TCGA COAD/READ, where representative regions are manually marked. In both cohorts, they show high prognostic value of eosinophils in the stroma. In contrast to our study, they also demonstrate the combined significant contribution of stromal lymphocytes and eosinophils.

In our multi-cohort investigation, we show different distributions of immune cells depending on the cohort, and different distribution of immune cells within a single patient. While many previous investigations, based on pathologists’ manually-derived scores define a cutoff and binarize their results, our fully automatic analysis allows us to analyze the continuous abundance of the different cell types across cases. Moreover, within patient heterogeneity suggests, that while evaluating a single slide may be more representative than a selected region or TMA core, studies should consider the entire case for evaluation. In addition, our study also compares the CRC immune response to the adjacent normal mucosa, where we could not find any correlation of the immune response close to the tumor and the general inflammation of the normal colon.

Earlier studies consistently report correlation with less aggressive characteristics such as low pT, low pN, or lack of lymphatic invasion^10,27,28^ suggesting a high prevalence of eosinophils in more favorable prognosis patients. Our study confirms these findings, in particular specifically for eosinophils in the tumor front. The low correlation of EosF with Immunoscore® on a subset of patients could also indicate added prognostic value on top of the Immunoscore®, but requires a larger investigation with complete Immunoscore® information.

Previous studies have extensively validated IELs (or often referred to as TILs or iTILs) and have shown a similar prognostic effect with hazard ratios below one^29–31^. Our study validates these findings with the exception of IELs in MSI cases, where we could not show a significant effect on outcome. In our analysis, IELs correlate with higher tumor grade which could suggest false positives, and HoVer-NeXt only achieves a moderate F1 score of 0.41 for their detection. In combination with the comparatively large confidence interval for IELs across survival analyses, this indicates that an improved deep learning model could potentially alter the results and lead to the same outcome as previous work has shown. Additionally, our method averages IEL prevalence over the entire slide while manual assessments often rely on the highest prevalence in one or more high powered fields.

Although not the focus of the current study, Crohn’s like lymphoid reaction (CLR) and tertiary lymphoid structures (TLS) also play an important role in the immune response. These structures, which would markedly alter the number of LymF, were excluded from this study which focused the investigation on eosinophils. While the Immunoscore®^4^ does not consider the presence of such TLS separately, other studies on CLR and TLS underline its important prognostic role^32,33^.

Experimental studies have been carried out to explain the prognostic relevance of eosinophils in the CRC TME, using molecular biology, mouse models and cell lines and highlight the direct or indirect anti-tumor activity of eosinophils^15–17,34–36^. Carretero et al.^35^, Arnold et al.^17^, and Reichman et al.^16^ all reporting anti-tumor activity of eosinophils against CRC cells, though only^16^ report a direct effect and Carretero et al.^35^ and Arnold et al.^17^ suggest a CD8+ lymphocyte recruitment as the anti-tumor measure. From our work, the combined prognostic value of IELs and EosF and lack of correlation could suggest that they result from separate molecular processes. On the other hand, LymF and EosF are correlated, which could be in line with the results of studies from Arnold et al.^17^ and Carretero et al.^35^ showing that eosinophils co-occur with CD8+ lymphocytes or even recruit them to the tumor site directly.

This study has some limitations. The threshold of 200*μm* for scoring could be discussed, with changes in this value also affecting results. Using slide-level normalization by tumor cell count in the respective front or center also includes information on the growth pattern and the density of tumor cells, which could also have an influence on the prognostic impact of the score. Finally, the retrospective nature of all cohorts in this study should also be considered when interpreting results.

Our study also has several strengths. We perform the to-date largest investigation into the prognostic value of eosinophils in CRC, with patient samples from multiple institutions. Moreover, our method is automatic, and reproducible and can therefore be easily adapted by other researchers or potentially translated to a diagnostic tool.

In summary, this study investigates the prognostic relevance of eosinophils in the CRC TME, while also accounting for lymphocytes, using a fully automated computational approach to separately investigate tumor front and tumor center. Our study provides further evidence that eosinophils may play a crucial role in anti-tumor response in CRC, independent of IELs, though potentially fulfilling multiple context-dependent roles. Moreover, our fully automatic approach to immune quantification is translatable to diagnostics without manual intervention.

Future work should further investigate eosinophils in the context of established immune scoring methods. Furthermore, single cell or single-cell spatial transcriptomics will give further insights into the phenotypes and expression profiles of eosinophils in CRC.

## Methods

### Patient Cohort Description

In this study, we utilize data from three different centers: University of Bern/ Inselspital Bern, Switzerland, RadboudUMC, Nijmegen, Netherlands, and Mount Sinai Hospital, Toronto, Canada. Additionally, we incorporate the publicly available TCGA COAD/READ dataset. All cohorts are retrospectively and independently collected. Individual approval was granted by the responsible ethics committee for each retrospectively collected pseudo anonymized cohort. The Bern cohort was approved by the Ethics committee of the Canton of Bern (reference number b2020-00498) in accordance with the Human Research Act HFG 2014. The Radboud and Toronto cohorts were approved by the ethics committee of Radboud University Medical Center (reference number 2015-1637) and Mount Sinai Hospital Research Ethics Board (reference number REB17-0054-E). Participation in these studies was based on an opt-out procedure or on informed consent, in agreement with ethical guidelines of the respective centers at the time of data collection. The study was conducted in accordance with the Declaration of Helsinki.

We exclude patients with neoadjuvant treatment, special morphologies such as signet ring cell or medullary carcinomas, and cases without available tumor tissue (Detailed exclusion criteria: Supp. Fig. S1). The combined cohort includes 1625 patients with 2529 Slides (Bern *N* = 195, 1084 Slides, Radboud *N* = 548, 548 Slides, Toronto *N* = 419, 423 Slides, TCGA COAD/READ *N* = 463, 468 Slides). Across cohorts tumor extension (pT), nodal status (pN), tumor location (right-sided, left-sided, location), treatment information, MSI, and histological subtype are available. For a subset of cohorts, venous (V), lymphatic (L), and perineural (Pn) invasion and tumor budding grade (BD)^37^ are available to investigate indicators of invasiveness. For a comparison with established immune response characterizations, data on the Immunoscore®^4^ (Radboud and Bern cohort) and Klintrup-Mäkinen score^2^ (Bern cohort), are available. Klintrup-Mäkinen score^2^ is a 4-tier evaluation of general inflammation in CRCs, particularly for the tumor front. The Immunoscore® is an IHC based scoring method of immune response based on the abundance of CD3 and CD8 positive T-cells in tumor front and center^38^. For TCGA COAD/READ, CMS, and PDS classification of tissue of the same case are available. The full dataset description can be found in supplementary Table S1. For the Bern cohort, we collect all primary tumor slides per patient. For the Toronto and Radboud cohorts, only the primary tumor slides with the deepest point of invasion were collected (Exceptions: four cases with two slides in Toronto, five cases with two slides in TCGA). All slides are formalin-fixed paraffin-embedded and H&E-stained in the respective institute of origin. They are digitized at 40x or ~0.24 microns per pixel (mpp). WSIs from both TCGA and Toronto cohorts were scanned using a Leica Aperio® AT2 scanner and the cohorts of Bern and Radboud using a 3DHistech Pannoramic 1000 scanner. For 52 patients from the Bern cohort, we additionally include a corresponding slide of tumor-free adjacent normal mucosa to compare the immune cell infiltration of the normal mucosa to the tumor within the same case.

### Deep Learning Pipeline

For this analysis, we build a pipeline which integrates several steps including two deep learning models to automatically quantify the immune response in CRC separately in tumor front and center.

**Fig. 6:**
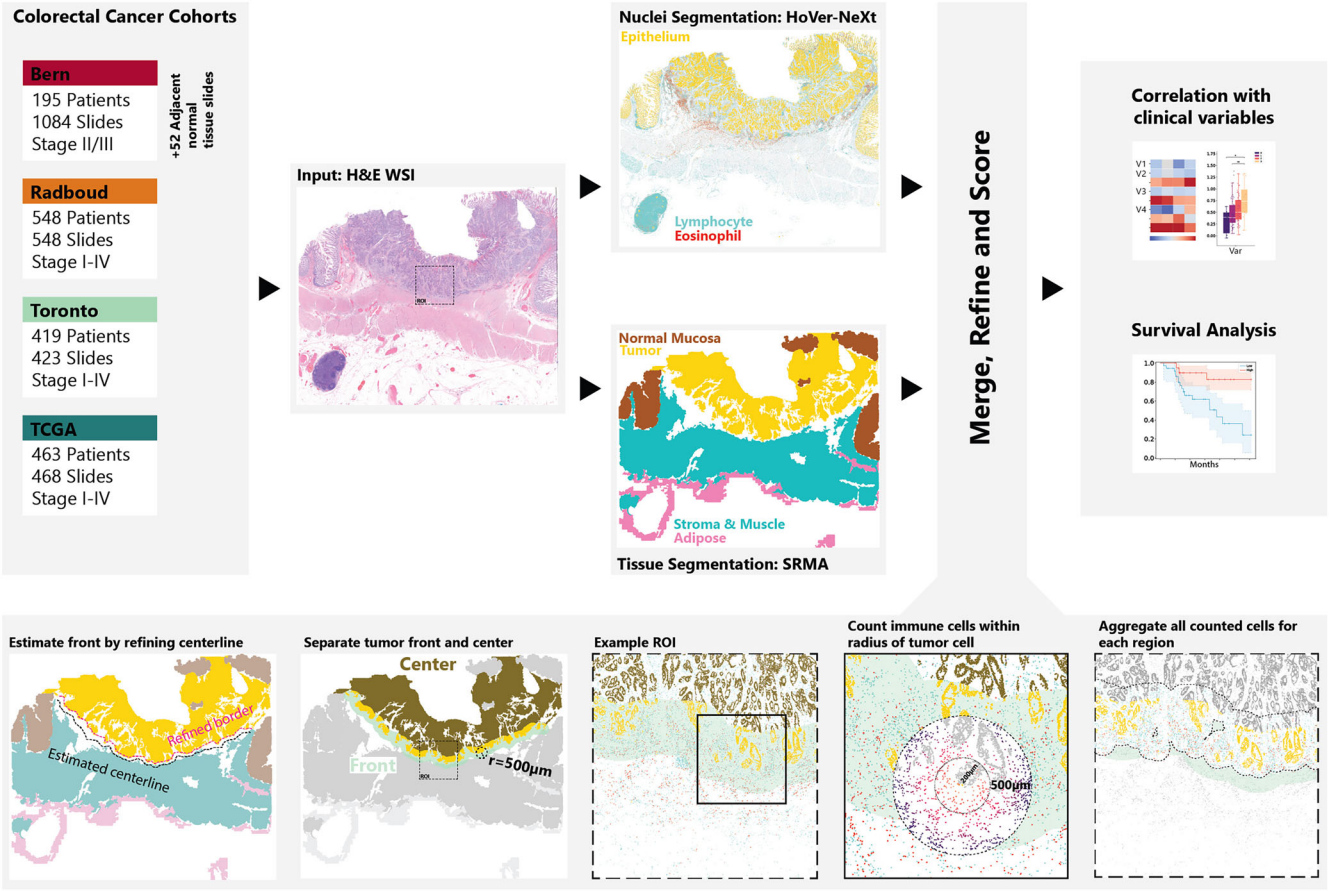
Project Overview with workflow and scoring method. Project workflow: the four cohorts with their H&-E-stained and scanned tissue slides are processed with two different deep learning algorithms. Algorithm results are refined, post-processed and scores are calculated. Finally, scores are used for downstream analysis including correlation with clinicopathological characteristics and survival analysis. Scoring workflow: First a tumor border is estimated, refined, and then used to separate tumor front and center. For each tumor cell in the respective region, all immune cells within a defined radius are counted and aggregated across all tumor cells in the region.

We use Self-Rule to Multi-Adapt (SRMA)^20^ with the coarse-to-refined adaptation^39^ for tissue type segmentation into nine classes: background, adipose, muscle, stroma, lymphoid aggregate, debris, normal epithelium, tumor epithelium, and mucin. For this analysis, we merge muscle and stroma and exclude the lymphoid aggregate class. We then use the segmented tissue map to locate the tumor on the slide, exclude any normal mucosa on the same slide and estimate the tumor front. Figure 6 illustrates this step in the workflow diagram where the initial tissue type segmentation is shown and subsequently reduced to the specific regions of interest.

For nuclei segmentation and classification on H&E-stained sections, we use HoVer-NeXt^19,40^ trained on Lizard-Mitosis. The model outputs nuclei panoptic segmentation maps for seven classes, epithelial, lymphocyte, neutrophil, plasma cell, eosinophil, mitosis and a catch-all category connective tissue cells. For this analysis, neutrophils, plasma cells, mitosis and connective tissue cells are all grouped as “other” cells. Eosinophils are a rare immune cell type compared to lymphocytes. To ensure that eosinophil detections are accurate, HoVer-NeXt is additionally validated on a holdout test set of 4952 manually annotated eosinophils. As HoVer-NeXt is a nuclei instance segmentation and classification model, we convert outputs to single points by using centroids and perform downstream analysis with coordinates only.

### Locating the Tumor Invasive Front and Tumor Center

To investigate immune populations at the tumor front and within the center separately, we first automatically estimate the tumor front using the tissue segmentation map. Instead of simply virtually drawing a margin around the entire tumor, we utilize the natural topology of CRCs, which grow into muscle and adipose tissue to find a separating line based on minimal distance between the tumor and these tissue types while omitting any area bordering the normal mucosa. Based on this line, we extend an area with 1000*μm* diameter. This means any tumor epithelial cells within 500*μm* of the estimated tumor front line are considered part of the tumor front^39^. Any detected tumor cells not in the tumor front are considered tumor center (Fig. 6).

### Intraepithelial Lymphocytes, Lymphoid Aggregates, and Lymph Nodes

To quantify intraepithelial lymphocytes (IELs), we define the epithelial tissue region by dilating tumor epithelial cells by 25*μm* and subtracting dilated (12.5*μm*) connective tissue cells, then count lymphocytes within this region. As the term tumor infiltrating lymphocyte is often used for a variety of different lymphocyte subsets^41^, we use “IELs”, when referring to lymphocytes that sit in between epithelial cells. In contrast, the term “lymphocyte” refers to lymphocytes in the stroma or other surrounding areas of the tumor.

To prevent lymphoid aggregates, tertiary lymphoid structures (TLS), and lymph nodes (LNs) from skewing lymphocyte counts, we identify these structures separately and exclude them from the analysis. We cluster lymphocytes by radius (20*μm*) and consider connected components of >500 lymphocytes as lymphoid aggregate, and >50000 lymphocytes as lymph nodes. Cutoffs are determined qualitatively using histogram analysis and visual inspection.

### Scoring

Immune cells are counted within 10*μm* radius bins around tumor cells up to 500*μm*. For survival analysis we accumulate counts in a fixed 200*μm* radius to quantify direct interactions between immune cells and tumor cells, but at the same time avoid counting disease-unrelated cells (Fig. 6 bottom center). To compare counts across regions, slides and cases, we normalize with the number of tumor cells in the respective region. The score is therefore defined as: The number of immune cell type within 200*μm* of a tumor cell in a region, normalized by the number of tumor cells in that region. Immune cells cannot be counted twice. For readability, we use abbreviations for lymphocytes in the front (LymF), in the center (LymC) and irrespective of region (LymA). The same abbreviation style is used for eosinophils (EosF, EosC, EosA). To avoid problems with epithelial crypt misclassifications, for IELs we specifically refine the scoring by computing the ratio of tumor cells with adjacent intraepithelial lymphocytes to those without. For cases with more than one slide per patient, slide level scores are weighted by tumor cell count and then averaged for patient level statistics. Furthermore, in TCGA, many H&E WSI contain only a small part of the entire resection specimen and therefore a proper tumor front cannot be assessed. For region-specific analysis, only cases where a front was successfully detected are included (*N* = 1406/1625, see Fig. S1).

### Statistical Analysis

Spearman correlation (*ρ*) analysis is used for rank correlation between continuous variables to ignore potentially different scaling between variables. The ± symbol indicates standard deviation. Distribution comparisons between categories are compared using Mann-Whitney U non-parametric tests. *P*-Values below 0.05 are considered significant and visualized as: * < 0.05, ** < 0.01, *** < 0.001, **** < 0.0001. Distributions in ordinal groups are compared using Kendall tau B (*τ*). Survival plots are created using Kaplan-Meier (KM) estimators and unless specified otherwise, survival plots are shown as quartile splits based on the full cohort. *P*-values for ordinal KM plots are estimated with a univariate Cox proportional hazard model and a likelihood ratio test. For categorical KM plots, *p*-values are Wald-tests against baseline in the same univariate Cox proportional hazard model. Model assumptions are always verified. Multivariate analysis is also performed using Cox proportionate hazard models, where all covariates are always included, even when they are not significant in univariate analysis. For Cox proportionate hazard model results, we always report Hazard Ratio (HR), 95% confidence interval (CI), and the associated *p*-value. Model selection and comparison is done via AIC using published recommendations^42^. In survival analysis, Lymphocytes and Eosinophils are log-transformed to scale linearly and better fit a normal distribution for the Cox proportional hazard model. Multiple testing correction is not applied, as the conclusions are drawn from individual comparisons, rather than a global hypothesis that applies to all comparisons^43^. Statistics are computed using Python 3.9, and SciPy (1.13.1), and Cox models are computed and evaluated in R 4.4.1 using survival (3.7.0).

### Endpoints

In order to measure the effect of immune response on disease and likelihood of metastasis, for all survival analysis time to recurrence (TTR) is used as endpoint. TTR is defined as the period from date of diagnosis to local or distant metastasis or cancer-specific death or end of follow-up for patients with no event. This endpoint ignores other reasons of death such as side-effects from therapy, old age or accidents which may be unrelated to the disease. As clinical data across cohorts is heterogeneous, a survival data cutoff of five years is set with patients having an event after five years being treated as no event at *T* = 60 months.

## Supplementary information

Supplementary Information

## Data Availability

Data generated in this study are available from the corresponding author (I.Z.) upon reasonable request. The eosinophil validation set is available at 10.5281/zenodo.11657620, the IEL validation set is available at 10.5281/zenodo.14998499.

## Code availability

The underlying code for this study and references to the used algorithms are available on github and can be accessed via this link: github.com/digitalpathologybern/CRC-Eos-Lym-IEL/.
