# Supplementary material for: Deep learning-based quantification of eosinophils and lymphocytes shows complementary prognostic effects in colorectal cancer patients
Source: NPJ Precis Oncol. 2025 Jun 13;9:175. doi: 10.1038/s41698-025-00955-0 (PMC12166090; doi:10.1038/s41698-025-00955-0)
Supplement: Supplementary file 1 — Supplementary Information [file 41698_2025_955_MOESM1_ESM.pdf]

I Supplementary Information

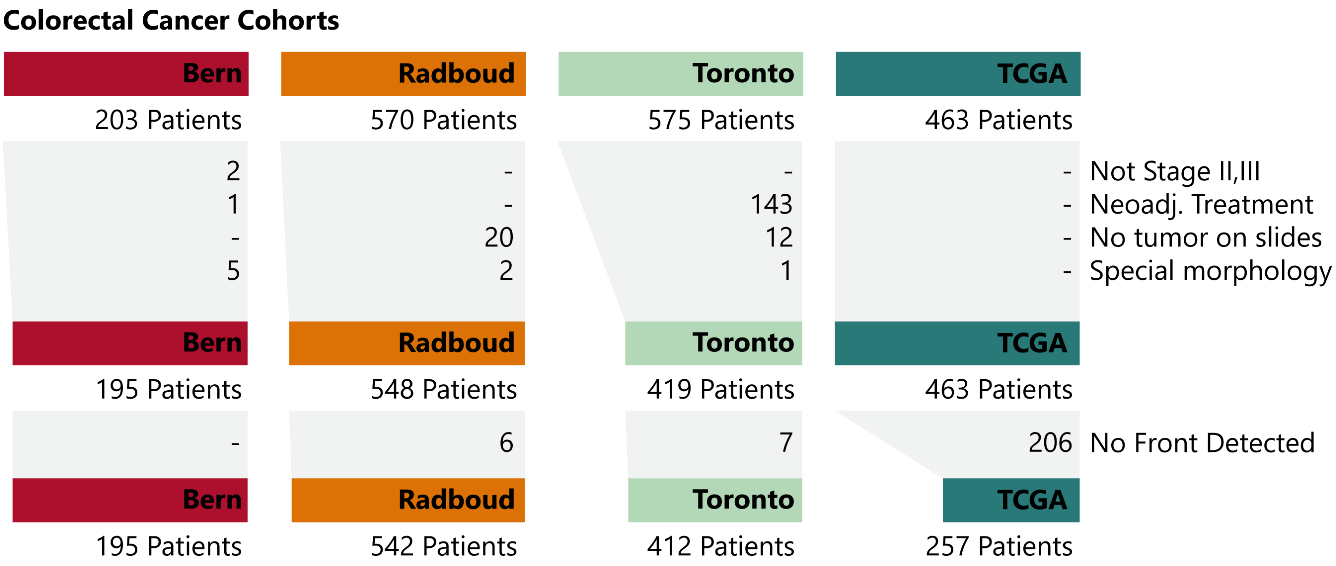

Figure S1. Exclusion criteria for the different cohorts.
Bern cohort should only include Stage II and III patients, but 2 patients do not fit this criterion according to current guidelines. For All cohorts, patients who received neoadjuvant treatment, without H&E slides with tumor, or patients with special morphologies such as neuroendocrine tumors are excluded. In a second step, slides were excluded if a tumor front could not be assessed.

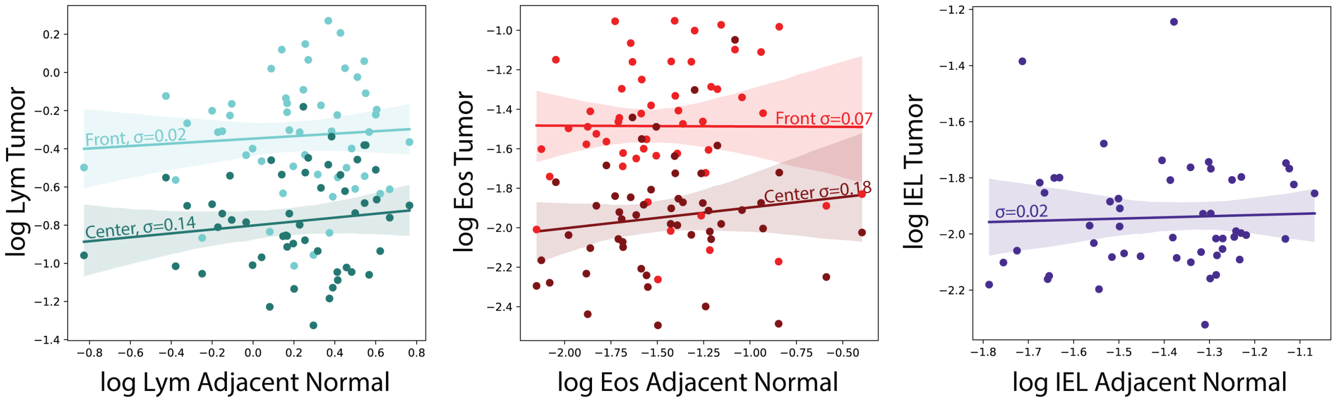

Figure S2. Correlation of immune scores for front and center with the respective estimated scores in the adjacent normal colon mucosa.
None of the comparisons show a strong correlation between normal mucosal abundances and the tumor microenvironment.

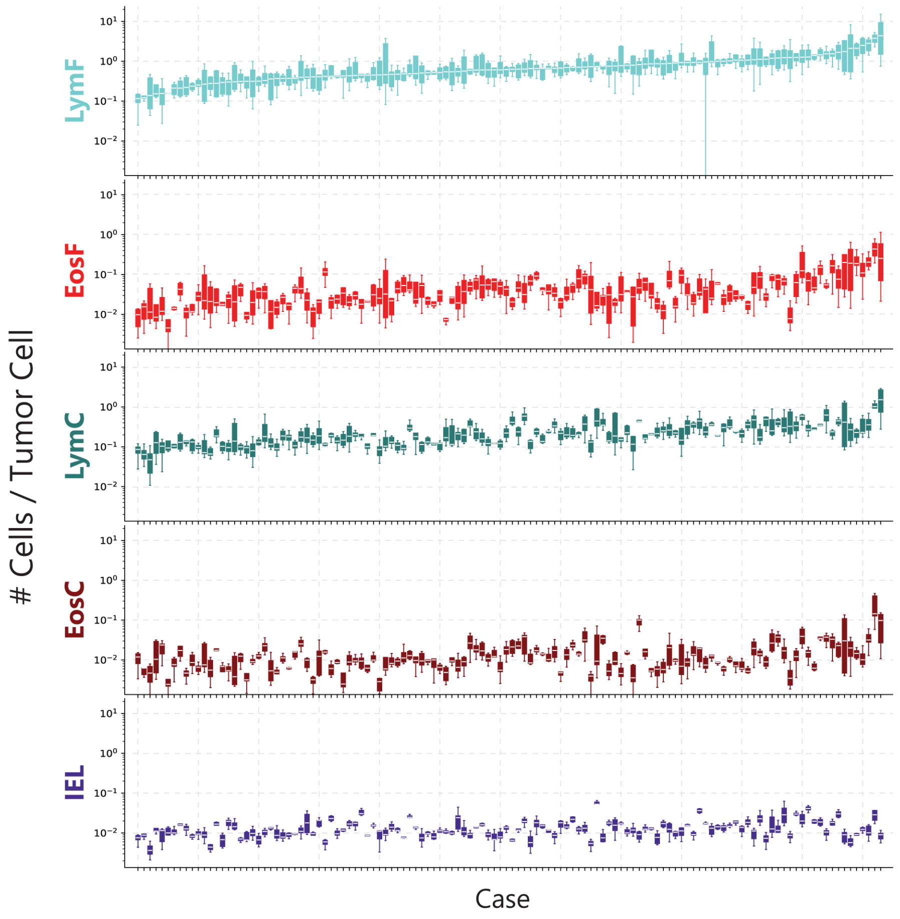

Figure S3. **Within case heterogeneity.**
Histogram of score distributions of cases from Bern cohort with at least five slides per case, sorted by median LymF in increasing order.

I.1 Cohort Table

		Bern		Radboud		Toronto		TCGA	
Patients		195		548		419		463	
slides		1084		548		423		474	
Mean Age	(min-max)	68.41	(19-92)	68.5	(27 - 93)	65.64	(22-94)	65.9	(31-90)
Sex	Female	82	42.1%	281	51.3%	197	47.0%	226	48.8%
	Male	113	57.9%	267	48.7%	222	53.0%	237	51.2%
Location	Right	99	50.8%	325	59.3%	153	36.5%	190	41.0%
	Left	93	47.7%	220	40.1%	193	46.1%	151	32.6%
	Rectum	3	1.5%	0	0.0%	73	17.4%	53	11.4%
pT	1	1	0.5%	10	1.8%	26	6.2%	14	3.0%
	2	8	4.1%	77	14.1%	77	18.4%	85	18.4%
	3	132	67.7%	338	61.7%	231	55.1%	311	67.2%
	4	54	27.7%	123	22.4%	85	20.3%	52	11.2%
pN	0	96	49.2%	235	42.9%	238	56.8%	266	57.5%
	1	69	35.4%	120	21.9%	126	30.1%	113	24.4%
	2	30	15.4%	82	15.0%	55	13.1%	83	17.9%
Adj. Chemotherapy	no	124	63.6%	430	78.5%	250	59.7%	293	63.3%
	yes	71	36.4%	105	19.2%	158	37.7%	170	36.7%
V	0	108	55.4%	-		171	40.8%	-	
	1	76	39.0%	-		248	59.2%	-	
L	0	70	35.9%	294	53.6%	157	37.5%	-	
	1	113	57.9%	128	23.4%	262	62.5%	-	
Pn	0	137	70.3%	363	66.2%	333	79.5%	-	
	1	33	16.9%	41	7.5%	86	20.5%	-	
Mucinous Histology	no	171	87.7%	415	75.7%	389	92.8%	403	87.0%
	yes	24	12.3%	131	23.9%	30	7.2%	60	13.0%
MSI	pMMR	155	79.5%	371	67.7%	365	87.1%	336	72.6%
	dMMR	34	17.4%	116	21.2%	50	11.9%	58	12.5%
Immunoscore (3T)	Low	26	13.3%	88	16.1%	-		-	
	Interm.	27	13.8%	224	40.9%	-		-	
	High	13	6.7%	110	20.1%	-		-	
Klintrup Makinen	0	7	3.6%	-		-		-	
	1	55	28.2%	-		-		-	
	2	63	32.3%	-		-		-	
	3	16	8.2%	-		-		-	
Budding	1	47	24.1%	-		163	38.9%	-	
	2	51	26.2%	-		117	27.9%	-	
	3	50	25.6%	-		137	32.7%	-	

Table S1. Clinicopathological characteristics for all cohorts. If values do not add up to number of patients, information is missing for some patients.

I.2 Supplementary Survival Analysis Tables

I.2.1 All Cases

		N	Univar HR	Lower CI	Upper CI	P	MV LymF HR	Lower CI	Upper CI	P	MV EosF HR	Lower CI	Upper CI	P	MV IEL HR	Lower CI	Upper CI	P
Age		1122	0.998	0.988	1.008	0.6569	0.9954	0.9847	1.0063	0.4087	0.995	0.9842	1.0058	0.3592	0.9972	0.9865	1.0081	0.6163
Sex	Female	548																
	Male	574	1.275	0.982	1.657	0.0687	1.3069	1.0023	1.7041	0.0481	1.278	0.9816	1.6639	0.0685	1.278	0.9814	1.6643	0.0687
Location	Left	678																
	Right	444	1.178	0.907	1.53	0.2191	0.8805	0.6707	1.1559	0.3595	0.8768	0.6678	1.1513	0.3441	0.907	0.6921	1.1887	0.4795
pT	I/II	185																
	III	721	3.48	1.883	6.43	0.0001	2.534	1.3572	4.7311	0.0035	2.5021	1.3391	4.6752	0.004	2.6262	1.4087	4.8958	0.0024
	IV	216	8.096	4.307	15.22	<0.0001	5.4045	2.8253	10.3382	<0.0001	5.1011	2.661	9.7788	<0.0001	5.0653	2.6354	9.7357	<0.0001
pN	0	620																
	I	327	2.529	1.838	3.48	<0.0001	2.0096	1.4089	2.8665	0.0001	1.9943	1.3987	2.8436	0.0001	2.0776	1.4563	2.9638	0.0001
	II	175	5.291	3.825	7.32	<0.0001	3.8946	2.7326	5.5508	<0.0001	3.7473	2.6252	5.3488	<0.0001	3.9929	2.7994	5.695	<0.0001
adj. Chemo	no	751																
	yes	371	1.707	1.315	2.217	0.0001	0.9188	0.6737	1.2531	0.5927	0.914	0.6709	1.2452	0.5687	0.9044	0.6629	1.2339	0.5261
MSI	no	905																
	yes	217	0.465	0.307	0.705	0.0003	0.5581	0.36	0.8652	0.0091	0.5513	0.3563	0.8531	0.0075	0.5998	0.3803	0.946	0.0279
LymF		1122	0.641	0.505	0.812	0.0002	0.7138	0.551	0.9248	0.0107								
LymC		1122	1.105	0.886	1.378	0.3773												
LymA		1122	1.12	0.906	1.384	0.2966												
EosF		1122	0.538	0.423	0.683	<0.0001					0.7023	0.5504	0.8961	0.0045				
EosC		1122	0.787	0.615	1.005	0.0552												
EosA		1122	0.78	0.622	0.977	0.0306												
IEL		1122	0.335	0.204	0.549	<0.0001									0.5831	0.3461	0.9824	0.0427

Table S2. Univariate and multivariate survival analysis using Cox proportional hazard model with TTR as dependent variable. All cases are included. First all variables are evaluated in univariate analysis. Then for each immune group, multivariate analysis is performed using all other variables and the single immune score.

		N	MV Lym,IEL HR	Lower CI	Upper CI	P	MV Lym,Eos HR	Lower CI	Upper CI	P	MV Eos,IEL HR	Lower CI	Upper CI	P	MV All HR	Lower CI	Upper CI	P
Age		1122	0.9959	0.9851	1.0068	0.4629	0.9949	0.9842	1.0057	0.354	0.9954	0.9846	1.0063	0.4066	0.9954	0.9846	1.0063	0.403
Sex	Female	548																
	Male	574	1.317	1.0097	1.7176	0.0422	1.2916	0.9903	1.6847	0.0591	1.2941	0.9935	1.6857	0.056	1.2993	0.9959	1.6951	0.0536
Location	Left	678																
	Right	444	0.8819	0.672	1.1574	0.3648	0.8736	0.6652	1.1472	0.3311	0.8762	0.6676	1.1499	0.3406	0.8749	0.6665	1.1485	0.3357
pT	I/II	185																
	III	721	2.4773	1.3264	4.6269	0.0044	2.4868	1.3309	4.6467	0.0043	2.4197	1.2945	4.5231	0.0056	2.416	1.2924	4.5161	0.0057
	IV	216	5.0419	2.6233	9.6903	<0.0001	5.167	2.6933	9.9126	<0.0001	4.6961	2.4387	9.0431	<0.0001	4.7317	2.4521	9.1306	<0.0001
pN	0	620																
	I	327	2.0149	1.4119	2.8753	0.0001	1.9874	1.3935	2.8344	0.0001	1.9906	1.3954	2.8395	0.0001	1.9881	1.3935	2.8364	0.0002
	II	175	3.9034	2.737	5.5668	<0.0001	3.7675	2.6382	5.3804	<0.0001	3.7377	2.6165	5.3393	<0.0001	3.7477	2.6221	5.3565	<0.0001
adj. Chemo	no	751																
	yes	371	0.9234	0.6765	1.2604	0.6156	0.9185	0.6738	1.252	0.5907	0.9247	0.678	1.2611	0.6208	0.926	0.6788	1.2632	0.6275
MSI	no	905																
	yes	217	0.63	0.3983	0.9966	0.0483	0.5602	0.3611	0.8689	0.0097	0.6458	0.4073	1.024	0.063	0.6466	0.4078	1.0252	0.0637
LymF		1122	0.7411	0.5716	0.961	0.0238	0.8772	0.6073	1.267	0.4848					0.9483	0.6538	1.3756	0.7799
LymC		1122																
LymA		1122																
EosF		1122					0.7659	0.5433	1.0797	0.1279	0.7052	0.5528	0.8997	0.0049	0.7306	0.5165	1.0333	0.076
EosC		1122																
EosA		1122																
IEL		1122	0.6397	0.3782	1.0822	0.0958					0.5924	0.3531	0.9941	0.0475	0.6013	0.3546	1.0195	0.059

Table S3. Multivariate survival analysis using Cox proportional hazard model with TTR as dependent variable. All cases are included and all combinations of immune scores are evaluated.

I.2.2 Stage II Cases

		N	Univar HR	Lower CI	Upper CI	P	MV Lym HR	Lower CI	Upper CI	P	MV Eos HR	Lower CI	Upper CI	P	MV IEL HR	Lower CI	Upper CI	P
Age		473	0.994	0.975	1.014	0.5809	0.9926	0.9728	1.0128	0.4674	0.9939	0.9743	1.0138	0.5434	0.9928	0.9727	1.0133	0.4897
Sex	Female	232																
	Male	241	1.758	1.055	2.929	0.0304	1.5923	0.9456	2.6811	0.0802	1.5256	0.9056	2.5701	0.1125	1.5569	0.9258	2.6184	0.0951
Location	Left	298																
	Right	175	1.697	1.039	2.771	0.0345	1.3992	0.8461	2.314	0.1906	1.3968	0.8455	2.3076	0.192	1.4349	0.8683	2.3711	0.1589
pT	III	394																
	IV	79	2.2	1.262	3.834	0.0054	2.7389	1.5552	4.8235	0.0005	2.5441	1.4486	4.4678	0.0012	2.4929	1.4154	4.3906	0.0016
MSI	no	350																
	yes	123	0.319	0.146	0.7	0.0044	0.3777	0.1667	0.8557	0.0196	0.3622	0.1605	0.8173	0.0145	0.4342	0.1866	1.0106	0.0529
LymF		473	0.621	0.396	0.975	0.0385	0.6449	0.4024	1.0334	0.0682								
LymC		473	0.892	0.575	1.386	0.6123												
LymA		473	0.966	0.638	1.464	0.8712												
EosF		473	0.608	0.391	0.947	0.0279					0.6744	0.4312	1.0548	0.0843				
EosC		473	0.728	0.459	1.154	0.1769												
EosA		473	0.759	0.497	1.158	0.2011												
IEL		473	0.247	0.092	0.666	0.0057									0.4006	0.139	1.1546	0.0903

Table S4. Univariate and multivariate survival analysis using Cox proportional hazard model with TTR as dependent variable. Only stage II cases are included. First all variables are evaluated in univariate analysis. Then for each immune group, multivariate analysis is performed using all other variables and the single immune score.

		N	MV Lym,IEL HR	Lower CI	Upper CI	P	MV Lym,Eos HR	Lower CI	Upper CI	P	MV Eos,IEL HR	Lower CI	Upper CI	P	MV All HR	Lower CI	Upper CI	P
Age		473	0.9912	0.9712	1.0117	0.3985	0.9928	0.9731	1.013	0.483	0.9919	0.972	1.0122	0.4319	0.9915	0.9716	1.0119	0.4114
Sex	Female	232																
	Male	241	1.5851	0.9416	2.6682	0.083	1.5614	0.9243	2.6377	0.0958	1.5262	0.9065	2.5697	0.1117	1.5459	0.9153	2.6108	0.1033
Location	Left	298																
	Right	175	1.3952	0.8431	2.3088	0.195	1.3894	0.8403	2.2973	0.1999	1.3871	0.839	2.2932	0.202	1.3825	0.8358	2.287	0.2072
pT	III	394																
	IV	79	2.5977	1.4692	4.593	0.001	2.6596	1.5006	4.7136	0.0008	2.4159	1.3716	4.2553	0.0023	2.4827	1.3914	4.43	0.0021
MSI	no	350																
	yes	123	0.443	0.19	1.033	0.0594	0.3748	0.1653	0.8499	0.0188	0.4458	0.1908	1.0417	0.0621	0.4463	0.1911	1.0425	0.0624
LymF		473	0.7004	0.434	1.1303	0.1448	0.7543	0.3936	1.4455	0.3955					0.8627	0.4455	1.6707	0.6615
LymC		473																
LymA		473																
EosF		473					0.8109	0.4404	1.4929	0.5008	0.6908	0.4412	1.0816	0.1058	0.7605	0.4103	1.4096	0.3845
EosC		473																
EosA		473																
IEL		473	0.4854	0.166	1.4199	0.1869					0.4302	0.1512	1.2239	0.1138	0.4568	0.1555	1.3418	0.1541

Table S5. Multivariate survival analysis using Cox proportional hazard model with TTR as dependent variable. Only stage II cases are included and all combinations of immune scores are evaluated.

I.2.3 MSI Cases

		N	Univar HR	Lower CI	Upper CI	P	MV Lym HR	Lower CI	Upper CI	P	MV Eos HR	Lower CI	Upper CI	P	MV IEL HR	Lower CI	Upper CI	P
Age		217	1.004	0.976	1.032	0.8002	1.0168	0.9838	1.0508	0.3229	1.0115	0.9777	1.0465	0.51	1.0149	0.9824	1.0484	0.3734
Sex	Female	136																
	Male	81	0.616	0.257	1.474	0.2761	0.8543	0.3146	2.3197	0.7573	0.821	0.3076	2.1916	0.6938	0.6496	0.2495	1.6915	0.377
Location	Left	188																
	Right	29	1.451	0.544	3.867	0.4573	1.5336	0.5113	4.5994	0.4454	1.7943	0.5801	5.55	0.3102	1.6682	0.5564	5.0019	0.361
pT	I/II	33																
	III	138	3.491	0.459	26.55	0.2272	1.4431	0.1765	11.802	0.7323	1.268	0.1534	10.4795	0.8256	1.8075	0.2269	14.3958	0.5761
	IV	46	7.357	0.941	57.485	0.0571	2.4998	0.2914	21.4467	0.4034	2.0331	0.2336	17.6914	0.5204	3.2814	0.3955	27.2264	0.271
pN	0	153																
	I	38	3.297	1.144	9.504	0.0272	2.4532	0.7838	7.6788	0.1232	2.7529	0.874	8.6706	0.0836	2.511	0.8154	7.7322	0.1086
	II	26	11.224	4.502	27.984	<0.0001	8.8135	3.2263	24.0768	0	9.409	3.4097	25.964	0	8.1946	2.9804	22.5308	0
adj. Chemo	no	168																
	yes	49	2.878	1.306	6.342	0.0087	1.7572	0.6457	4.7817	0.2697	1.6513	0.6073	4.4902	0.3257	1.5655	0.5802	4.2244	0.3762
LymF		217	0.499	0.239	1.044	0.0648	0.4804	0.2226	1.0369	0.0618								
LymC		217	1.158	0.645	2.08	0.6227												
LymA		217	1.179	0.649	2.139	0.5892												
EosF		217	0.342	0.155	0.756	0.008					0.3217	0.1395	0.7418	0.0078				
EosC		217	0.545	0.258	1.152	0.1118												
EosA		217	0.546	0.269	1.108	0.0936												
IEL		217	0.72	0.262	1.977	0.5238									0.9271	0.31	2.7725	0.8923

Table S6. Univariate and multivariate survival analysis using cox proportional hazard model with TTR as dependent variable. Only MSI cases are included. First all variables are evaluated in univariate analysis. Then for each immune group, multivariate analysis is performed using all other variables and the single immune score.

		N	MV Lym,IEL HR	Lower CI	Upper CI	P	MV Lym,Eos HR	Lower CI	Upper CI	P	MV Eos,IEL HR	Lower CI	Upper CI	P	MV All HR	Lower CI	Upper CI	P
Age		217	1.016	0.9828	1.0504	0.3482	1.0111	0.9767	1.0466	0.5325	1.0124	0.9778	1.0482	0.4876	1.0118	0.977	1.0479	0.5107
Sex	Female	136																
	Male	81	0.8575	0.3158	2.3287	0.763	0.8077	0.2951	2.2107	0.6776	0.8272	0.3089	2.2152	0.7058	0.8012	0.2916	2.201	0.6673
Location	Left	188																
	Right	29	1.5599	0.5174	4.7029	0.4297	1.8068	0.5816	5.6129	0.3064	1.7529	0.5589	5.4978	0.3359	1.7584	0.5594	5.5274	0.3341
pT	I/II	33																
	III	138	1.4741	0.1783	12.1898	0.7188	1.2735	0.1541	10.527	0.8225	1.229	0.1479	10.2121	0.8486	1.2279	0.1487	10.1426	0.8488
	IV	46	2.573	0.2949	22.4472	0.3925	2.039	0.2345	17.7285	0.5185	1.9601	0.2233	17.2087	0.5437	1.9473	0.2234	16.9728	0.5463
pN	0	153																
	I	38	2.4637	0.788	7.7028	0.1211	2.7689	0.8779	8.7328	0.0823	2.7375	0.8665	8.6483	0.0862	2.7678	0.8756	8.7495	0.083
	II	26	8.962	3.2582	24.651	<0.0001	9.4066	3.4088	25.9571	<0.0001	9.2461	3.3227	25.7297	<0.0001	9.1594	3.2822	25.5607	<0.0001
adj. Chemo	no	168																
	yes	49	1.7366	0.6374	4.7315	0.2805	1.6408	0.6017	4.4741	0.3333	1.6862	0.6113	4.6511	0.3128	1.6805	0.6091	4.6363	0.3161
LymF		217	0.4662	0.2075	1.0475	0.0646	1.0797	0.3812	3.0581	0.8852					1.1816	0.3788	3.6858	0.7737
LymC		217																
LymA		217																
EosF		217					0.3026	0.0927	0.9876	0.0476	0.3232	0.1411	0.7399	0.0075	0.2831	0.082	0.977	0.0458
EosC		217																
EosA		217																
IEL		217	1.1639	0.3772	3.5919	0.7918					0.8622	0.2993	2.4832	0.7835	0.8036	0.252	2.5623	0.7117

Table S7. Multivariate survival analysis using Cox proportional hazard model with TTR as dependent variable. Only MSI cases are included and all combinations of immune scores are evaluated.

I.2.4 MSS Cases

		N	Univar HR	Lower CI	Upper CI	P	MV Lym HR	Lower CI	Upper CI	P	MV Eos HR	Lower CI	Upper CI	P	MV IEL HR	Lower CI	Upper CI	P
Age		905	0.9976	0.9866	1.0087	0.6671	*0.9913*	0.9795	1.0032	0.1495	0.9914	0.9797	1.0032	0.1533	0.9926	0.9808	1.0046	0.2269
Sex	Female	412																
	Male	493	1.3091	0.9883	1.7339	0.0604	1.4252	1.0713	1.896	0.015	1.3913	1.0475	1.848	0.0226	1.4122	1.062	1.878	0.0176
Location	Left	490																
	Right	415	1.0164	0.7718	1.3386	0.9077	0.8492	0.642	1.1234	0.2522	0.8479	0.641	1.1217	0.2479	0.8737	0.6616	1.1536	0.3409
pT	I/II	152																
	III	583	3.5822	1.881	6.8223	0.0001	2.686	1.3952	5.1712	0.0031	2.6761	1.3891	5.1555	0.0033	2.7468	1.4287	5.281	0.0024
	IV	170	8.982	4.6225	17.4528	<0.0001	5.9934	3.0296	11.8564	<0.0001	5.6963	2.8731	11.2939	<0.0001	5.4505	2.7366	10.8556	<0.0001
pN	0	467																
	I	289	2.2782	1.6286	3.1871	<0.0001	1.9633	1.3494	2.8564	0.0004	1.9486	1.3395	2.8345	0.0005	2.0128	1.3844	2.9266	0.0002
	II	149	4.4836	3.1672	6.3472	<0.0001	3.5155	2.4075	5.1335	<0.0001	3.4074	2.3284	4.9864	<0.0001	3.6277	2.4835	5.2992	<0.0001
adj. Chemo	no	583																
	yes	322	1.5376	1.1661	2.0275	0.0023	0.8507	0.6119	1.1825	0.3359	0.8506	0.6123	1.1816	0.3345	0.8416	0.6053	1.1701	0.305
LymF		905	0.7195	0.5592	0.9258	0.0105	0.7388	0.5614	0.9722	0.0307								
LymC		905	1.2372	0.9669	1.5829	0.0906												
LymA		905	1.2168	0.9678	1.53	0.0930												
EosF		905	0.6	0.4673	0.7704	0.0001					0.7512	0.5828	0.9682	0.0271				
EosC		905	0.8587	0.6621	1.1139	0.2512												
EosA		905	0.8528	0.672	1.0822	0.1902												
IEL		905	0.3464	0.1899	0.632	0.0005									0.5132	0.2808	0.9379	0.0301

Table S8. Univariate and multivariate survival analysis using cox proportional hazard model with TTR as dependent variable. Only MSS cases are included. First all variables are evaluated in univariate analysis. Then for each immune group, multivariate analysis is performed using all other variables and the single immune score.

		N	MV Lym,IEL HR	Lower CI	Upper CI	P	MV Lym,Eos HR	Lower CI	Upper CI	P	MV Eos,IEL HR	Lower CI	Upper CI	P	MV All HR	Lower CI	Upper CI	P
Age		905	0.9914	0.9795	1.0034	0.1584	*0.9911*	0.9794	1.003	0.1423	0.9914	0.9796	1.0033	0.1562	0.9912	0.9794	1.0032	0.1503
Sex	Female	412																
	Male	493	1.4427	1.0839	1.9203	0.012	1.4098	1.0592	1.8764	0.0186	1.4136	1.0634	1.8792	0.0171	1.4232	1.0687	1.8954	0.0158
Location	Left	490																
	Right	415	0.852	0.6443	1.1267	0.2612	0.8444	0.6381	1.1172	0.2363	0.8489	0.642	1.1225	0.2505	0.8469	0.6403	1.1202	0.2443
pT	I/II	152																
	III	583	2.6139	1.3571	5.0344	0.0041	2.6552	1.3783	5.115	0.0035	2.5806	1.339	4.9737	0.0046	2.5731	1.3351	4.9593	0.0048
	IV	170	5.4723	2.7486	10.8952	<0.0001	5.8037	2.9239	11.5201	<0.0001	5.1611	2.5876	10.294	<0.0001	5.2303	2.6156	10.4591	<0.0001
pN	0	467																
	I	289	1.9571	1.3455	2.8468	0.0004	1.9423	1.3349	2.8262	0.0005	1.9313	1.3279	2.8087	0.0006	1.9294	1.3264	2.8065	0.0006
	II	149	3.5336	2.4182	5.1636	<0.0001	3.4288	2.3415	5.0208	<0.0001	3.4066	2.3255	4.9902	<0.0001	3.421	2.3339	5.0145	<0.0001
adj. Chemo	no	583																
	yes	322	0.8536	0.6139	1.187	0.3468	0.8531	0.6139	1.1855	0.344	0.8577	0.6173	1.1917	0.3603	0.8584	0.6177	1.1929	0.3632
LymF		905	0.7704	0.585	1.0145	0.0633	0.8521	0.5733	1.2665	0.4288					0.9179	0.6164	1.3667	0.673
LymC		905																
LymA		905																
EosF		905					0.835	0.5801	1.2019	0.3319	0.758	0.5874	0.978	0.0331	0.8023	0.5561	1.1576	0.239
EosC		905																
EosA		905																
IEL		905	0.5574	0.304	1.0223	0.0589					0.5273	0.2893	0.9611	0.0367	0.5387	0.2932	0.9895	0.0462

Table S9. Multivariate survival analysis using Cox proportional hazard model with TTR as dependent variable. Only MSI cases are included and all combinations of immune scores are evaluated.
